# The association between FOXO3a rs4946936 gene polymorphism and the levels of FOXO3a among chronic granulocytic leukemia patients treated with imatinib mesylate

**DOI:** 10.12688/f1000research.73054.3

**Published:** 2022-04-11

**Authors:** Shinta Oktya Wardhani, Hani Susianti, Puji Rahayu, Yuyun Prabowowati Yueniwati, Jonny Karunia Fajar

**Affiliations:** 1 Division of Hematology and Oncology, Department of Internal Medicine, Faculty of Medicine, Universitas Brawijaya, Malang, East Java, 65145, Indonesia; 2 Department of Clinical Pathology and Laboratory Medicine, Faculty of Medicine, Universitas Brawijaya, Malang, East Java, 65145, Indonesia; 3 Department of Otorhinolaryngology, Faculty of Medicine, Universitas Brawijaya, Malang, East Java, 65145, Indonesia; 4 Department of Radiology, Faculty of Medicine,, Universitas Brawijaya, Malang, East Java, 65145, Indonesia; 5 Brawijaya Internal Medicine Research Center, Department of Internal Medicine, Faculty of Medicine, Universitas Brawijaya, Malang, East Java, 65145, Indonesia

**Keywords:** chronic granulocytic leukemia, FOXO3a, FOXO3a rs4946936 gene polymorphism

## Abstract

**Background: **The gene *FOXO3a* has been elucidated to govern the development of chronic granulocytic leukemia (CGL). Moreover, it has been suggested that the levels of *FOXO3a* in circulation are affected by the *FOXO3a* rs4946936 gene polymorphism. However, no study has assessed the correlation between the *FOXO3a* rs4946936 gene polymorphism and the levels of *FOXO3a*. The objective of this study was to assess the association between the *FOXO3a* rs4946936 gene polymorphism and the levels of *FOXO3a* in CGL patients treated with imatinib mesylate.

**Methods:** A cross-sectional study was conducted from February 2019 to February 2020. The genotyping of *FOXO3a* rs4946936 gene polymorphism was conducted using PCR-RFLP, and the levels of *FOXO3a* were assessed using ELISA. The association between the *FOXO3a* rs4946936 gene polymorphism and the levels of *FOXO3a* were assessed using multiple logistic regression.

**Results:** A total of 60 CGL patients were assessed in our study. Among them, the CC, CT, and TT genotypes of the *FOXO3a* rs4946936 gene polymorphism were 35.0%, 48.3%, and 16.7% respectively. Our calculation revealed that elevated levels of *FOXO3a* were found in CGL patients with the CC genotype of the *FOXO3a* rs4946936 gene polymorphism. While we failed to clarify the association between either the CT or the TT genotype of *FOXO3a* rs4946936 gene polymorphism and the levels of *FOXO3a*.

**Conclusion:** Our study identifies that the CC genotype of the *FOXO3a* rs4946936 gene polymorphism affects the elevated levels of *FOXO3a* in CGL patients treated with imatinib mesylate.

## Introduction

Chronic granulocytic leukemia (CGL), first recognized in 1845, is a myeloproliferative disease caused by a condition in which a single pluripotential haemopoetic stem cell acquires the Philadelphia chromosome. ^1^ A global report in 2015 revealed that the incidence of this disease was estimated between 0.7 and 1.0 per 100,000 inhabitants, ^2^ while mortality was reported at less than 15%. ^3^ The pathogenesis of CGL is a comprehensive process, and may involve a wide variety of biomarkers including cyclooxgenase 1 ( *COX1*), patched homolog 1 ( *PTCH1*), Forkhead Transcription Factor 3a ( *FOXO3a*), prostaglandin-endoperoxide synthase 1 ( *PTGS1*), and human organic cation transporter 1 ( *hOCT1*). ^4^^–^^6^ Of them, *FOXO3a* is proposed as one biomarker having a pertinent role in the pathogenesis of CGL, although evidence is limited. ^6^

*FOXO3a* belongs to the FOXO Forkhead transcription factors subfamily. The FOXO transcription factors have a various function in the cell stability, including the activation of target – gene expression and the inhibition of target – gene expression, depending on the type of their subfamily and their interaction to the specific protein. ^7^ The subfamily of FOXO includes *FOXO1*, *FOXO3a*, *FOXO3b*, *FOXO4* and *FOXO6.* ^8^ Of them, *FOXO3a* palys a dominant role to activate the expression of target gene in the pathogenesis of CGL, and may contribute to the progression of CGL. ^6^^,^^7^^,^^9^ Moreover, the existence of *FOXO3a* in the circulation is governed by the *FOXO3a* gene, which has several single nucleotide polymorphisms (SNPs) such as rs2802292, rs2764264, rs4945816, rs9400239, rs4946936, and rs13217795. ^10^ Of them, the *FOXO3a* rs4946936 gene variant is suggested to have a crucial role in affecting the levels of *FOXO3a* in circulation, and therefore may have a potential role in the pathogenesis of CGL. While the gene polymorphism of *FOXO3a* rs4946936 has been investigated in the case of vitiligo, ^11^ chronic obstructive pulmonary disease (COPD), ^12^ acute lymphoblastic leukemia, ^13^ thyroid cancer, ^14^ and hepatocellular carcinoma, ^15^ the role of *FOXO3a* rs4946936 gene polymorphisms in the development of CGL has never been examined. Therefore, we aimed to assess the correlation between *FOXO3a* rs4946936 gene polymorphism and the levels of *FOXO3a* in patients with CGL treated with imatinib mesylate. Our report may serve as an initial insight into the role of the *FOXO3a* rs4946936 gene polymorphism in the pathogenesis of CGL.

## Methods

### Study design and patients

During the period from February 2019 to February 2020, we conducted a cross-sectional study in Saiful Anwar General Hospital, Malang, Indonesia. A total sampling method was applied to recruit the study participants (a total of 26 participants was needed as the minimum sample size according to the estimation that the prevalence of CGL was 10–12 per 100,000 inhabitants with a 5% margin of error and 95% confidence level). The patients were included in our study if they met the following inclusion criteria: (1) all CGL patients (confirmed by a positive Bcr-ABL test) treated with imatinib mesylate for at least six months in our hospital during the study period, (2) aged more than 18 years old, and (3) willing to participate in the study, proven by giving written informed consent. Patients were excluded from our study if they had a history of the following conditions: prostate cancer, gynecological cancer, COPD, breast cancer, vitiligo, and thyroid cancer. ^16^ Our present study was conducted following the ethics code of the Helsinki Declaration and was registered and approved by the Ethical Committee of Universitas Brawijaya (No. 400/098/k.3/302/2019).

### Genotyping of *FOXO3a* rs4946936 gene polymorphism

We collected venous blood in plastic vacutainer tubes containing EDTA. We separated the blood samples, and they were stored at −20 °C. We centrifuged the blood samples in EDTA at 1000 ×g for 10 min, and we separated the plasma and stored it at −20 °C until it was used to measure *FOXO3a* levels. A polymerase chain reaction-restriction fragment length polymorphism (PCR-RFLP) method was applied to genotype the *FOXO3a* rs4946936 gene polymorphism. The procedures in *FOXO3a* genotyping including the primer used, amplification, and PCR cycles (initial denaturation, denaturation, annealing, and extension) were adapted from previous studies, ^11^^,^^12^ using the CFX96 TouchTM Real-Time PCR Detection System (Bio-Rad, California, USA). The *FOXO3a* primer ( *FOXO3a* F: 5′-GGGTCCTGAGAACTTCTGAGT-3′; *FOXO3a* R: 5′-GACATTCTGTAAGACATTCTGCCT-3′) used in our present study was MyTaq HS Mix, 2X (Bioline, Meridian BioscienceTM, OH, USA). We used SfcI (New England Biolabs, Massachusetts, USA) as the restriction enzyme. PCR was conducted in a 50 μl volume with 20–100 ng DNA, 20 pmol of each primer, 50 μM KCl, 100 μm dNTPs, 20 μM Tris-HCl pH 8.6.

**Figure 1.**
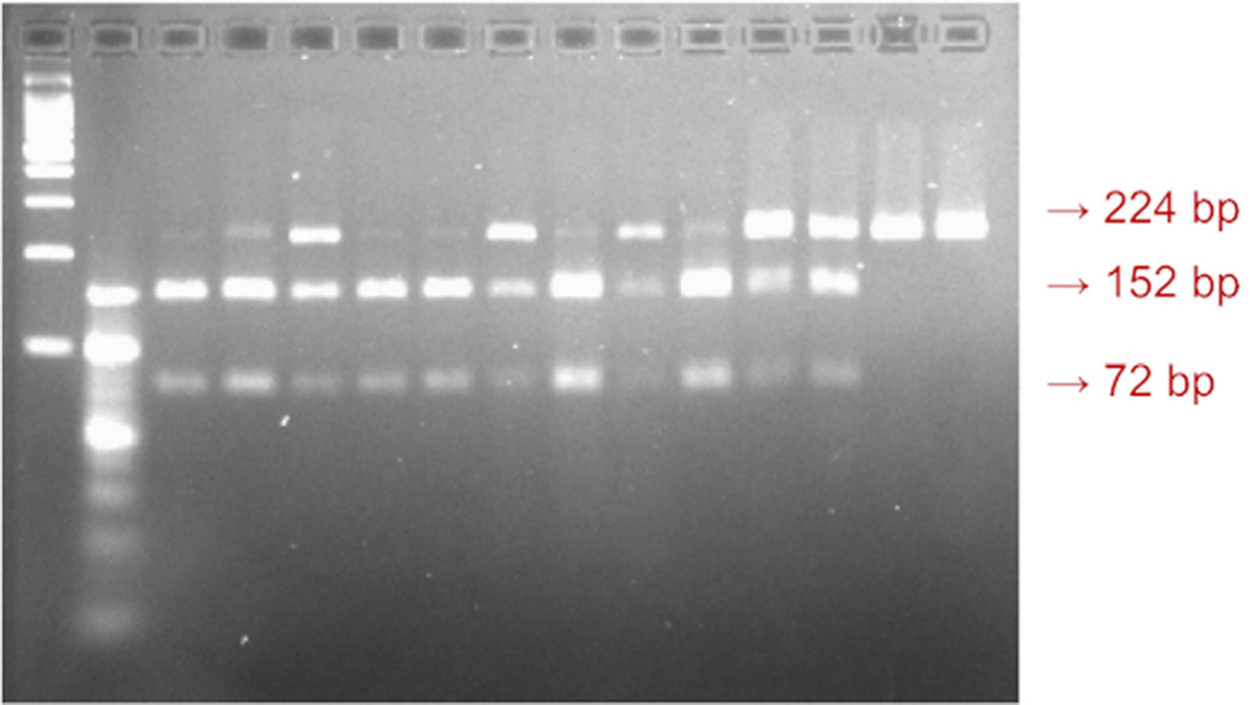
A visualization of the *FOXO3a* rs4946936 gene polymorphism. The 224bp sized band indicated TT genotype, the 152bp size band indicated CT genotype, and the 72bp sized band indicated CC genotype.

1 mM MgCl _2_, and 1 U Taq polymerase (MBI Fermentas, Vilnius, Lithuania). Amplification was carried out using an automated thermal cycler (Techne-Genius, Princeton, NJ). The PCR cycles consisted of initial denaturation at 94 °C for 3 min; 35 cycles of denaturation at 94 °C for 1 min, annealing at 53 °C for 1 min, and extension at 72 °C for 1 min, followed by final extension at 72 °C for 7 min. The amplified products were digested with one unit of Sfcl (rs4946936; C/T). The digested fragments were separated on 2% agarose gel by electrophoresis. The genotypes were identified according to their fragment sizes. The TT genotype was characterized by 224 bp sized bands, the TC genotype was characterized by 224, 152, and 72 bp sized bands, and the size bands of 152 and 72 indicated the CC genotype ( Figure 1).

### The measurement of *FOXO3a* levels

The measurement of serum *FOXO3a* levels was conducted by the enzyme-linked immunosorbent assay (ELISA) method, using the Cusabio kit (Cusabio Biotech Co., New York, USA), with the procedures conforming to the manual instructions from the company. The 100 μl standard liquid and blood sample were added to the tubes, and incubated for 90 min. The liquid from each tube was then shucked. The 100 μl biotinylated detection antibody was added to each tube and incubated at 37 °C for 1h. The liquid of each tube was shucked and washed three times. The 100 μl 1x HRP conjugate was then added to each tube and incubated at 37 °C for 30 min. The liquid was shucked and washed five times. The 90 μl TMB substrate was added to each tube and incubated at 37 °C for 15 min. The 50 μl stoper liquid was added to each tube. At this step, a change from blue to yellow might occur. Measurement of optic density at 450 nm was conducted. The results were then interpreted to the standard curve to determine the levels of *FOXO3a* and expressed in units of pg/mL.

### Statistical analysis

The correlation between the *FOXO3a* rs4946936 gene polymorphism and *FOXO3a* levels was assessed using multiple logistic regression. A p-value of less than 0.05 indicated a significant association. The effect estimates between groups were presented by calculation of the mean difference. We used the R stats package statistical software to analyze the data in our study (R Project for Statistical Computing, RRID:SCR_001905).

## Results

### Baseline characteristics

A total of 60 CGL patients were analyzed. Of them, 21 (35.0%), 29 (48.3%), and 10 (16.7%) patients had a CC, CT, and TT genotype, respectively. Initially, we employed a total of 68 CGL patients, however, eight patients were excluded due to having a history of gynecological cancer and COPD. Table 1 describes the baseline characteristics of patients included in our study. The detailed data are presented as underlying data. ^17^

**Table 1.** Baseline characteristics of chronic granulocytic leukemia patients treated with imatinib mesylate included in the study examining the association between *FOXO3a* rs4946936 gene polymorphism and the levels of *FOXO3a.*

Characteristics	*FOXO3a* gene polymorphism				p
	CC	CT	TT	Total	
Gender					
Male	11 (18.30)	15 (25.00)	5 (8.30)	31 (51.70)	0.992
Female	10 (16.70)	14 (23.30)	5 (8.30)	29 (48.30)	
Age	43.62 ± 11.75	41.9 ± 12.53	43.4 ± 14.18	10.12 ± 4.04	0.877
Spleen					
S3	4 (6.70)	4 (6.70)	0 (0.00)	8 (13.30)	0.455
S4	6 (10.00)	7 (11.70)	4 (6.70)	17 (28.30)	
S5	5 (8.30)	13 (21.70)	5 (8.30)	23 (38.30)	
S6	6 (10.00)	5 (8.30)	1 (1.70)	12 (20.00)	
Hemoglobin	11.59 ± 4.18	10.9 ± 3	10.12 ± 4.04	11.01 ± 3.6	0.559
Leucocyte	25698.1 ± 56533.51	42165.55 ± 94168.6	138092 ± 162617.05	52389.68 ± 104285.9	0.013
Thrombocyte	218047.62 ± 102640.38	423241.38 ± 963616.32	306900 ± 183752.52	332033.33 ± 676914.46	0.575

Note: Total no. of patients = 60. Data were presented as mean ± SD or no. (%).

### Main findings

**Figure 2.**
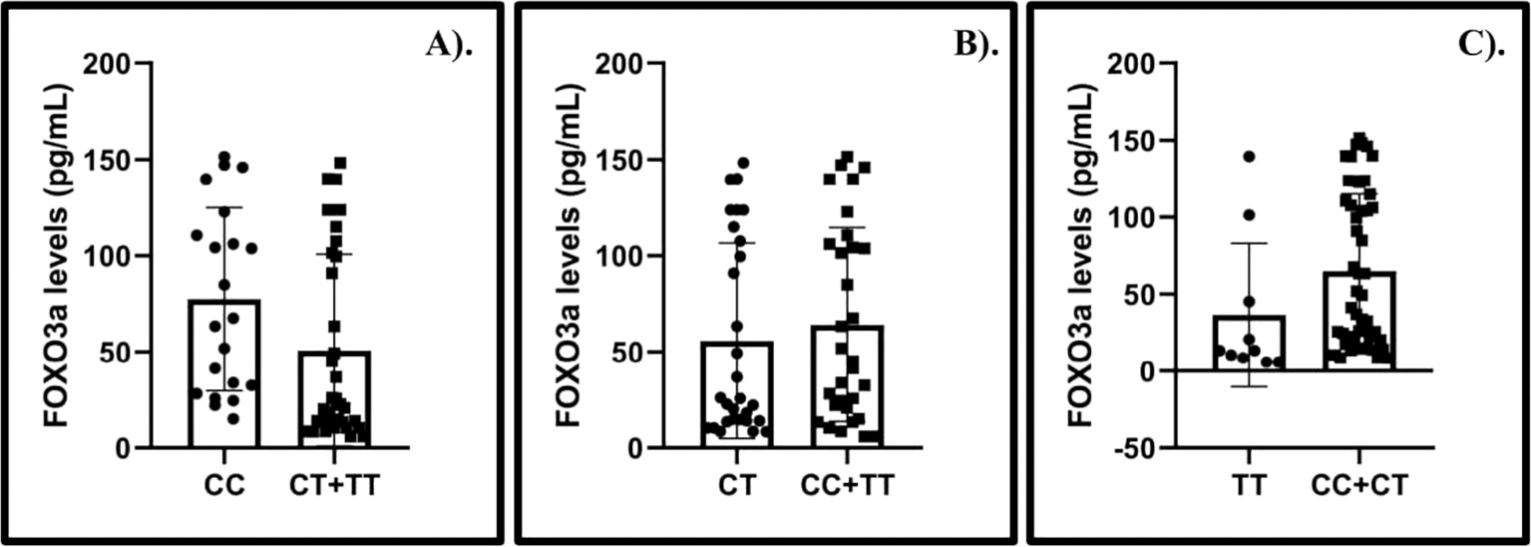
The summary of *FOXO3a* levels and the gene polymorphism of *FOXO3a* in chronic granulocytic leukemia patients treated with imatinib mesylate. A). CC vs. CT+TT, MD: 26.56, 95%CI: 0.48, 52.64, p: 0.0460; B). CT vs. CC+TT, MD: −8.45, 95%CI: −34.09, 17.19, p: 0.5180; C). TT vs. CC+CT, MD: −28.33, 95%CI: −62.06, 5.39, p: 0.1000.

Our findings identified that elevated levels of *FOXO3a* were observed among CGL patients with CC (CC vs. CT + TT) genotype (MD: 26.56; 95% CI: 0.48, 52.64). While the association between the CT (CT vs. CC + TT) and TT (TT vs. CC + CT) genotypes and the levels of *FOXO3a* were unverified ( Figure 2).

## Discussion

Our study identified that the CC genotype of the *FOXO3a* rs4946936 gene polymorphism was associated with increased levels of *FOXO3a* among patients with CGL treated with imatinib mesylate. The study in the context of the association between the *FOXO3a* rs4946936 gene polymorphism and the levels of *FOXO3a* in patients with CGL treated with imatinib mesylate had, to our knowledge, never been performed previously, therefore, an holistic comparison including the gene-ethnicity or gene-environment interaction could not be discussed. Previous studies in this context had been performed in the case of COPD ^12^ and vitiligo. ^11^ In the case of vitiligo, a study failed to clarify the correlation between the levels of *FOXO3a* and the gene polymorphism of *FOXO3a* rs4946936. ^11^ Moreover, in the case of COPD, no association was observed between the levels of *FOXO3a* and the gene polymorphism of *FOXO3a* rs4946936. ^12^ The different findings between our study in the case of CGL and previous studies in the case of vitiligo and COPD are contradictory and debatable, and a possible reason might be proposed. Briefly, vitiligo and COPD are known as conditions that affect the levels of *FOXO3a*, and the mechanism of *FOXO3a* production in the case of CGL might differ to the case of vitiligo and COPD. In the case of vitiligo, *FOXO3a* may interrupt the oxidative pathway by controlling the target gene expression. ^11^ In the case of COPD, *FOXO3a* may stimulate the expression of Atrogin-1/MAFbx, a biological marker having a pivotal role in the development of COPD. ^12^ On the other hand, the role of *FOXO3a* in the case of CGL occurs through establishing the phosphorylation by p210BCR-ABL tyrosine kinase, and may contribute to the proliferation of leukemic progenitors. ^18^^,^^19^

Theoretically, the association between the levels of *FOXO3a* and the gene polymorphism of *FOXO3a* rs4946936 remain conflicting. However, several mechanisms might be proposed. First, the gene-gene interaction or gene-protein interaction might underly our findings. This possible mechanism was supported by a previous study. ^20^ The authors revealed that a dominant homozygote genotype of the *FOXO3a* rs4946936 gene variant was reported to associate with elevated levels of immunoglobulin (Ig) E in the case of asthma. They proposed that *FOXO3a* might regulate the production of proinflammatory cytokine, and cause elevated levels of IgE. ^20^ Second, free energy levels between genotypes of the *FOXO3a* rs4946936 gene polymorphism might affect the levels of *FOXO3a* in the circulation. In an *in silico* study, the free energy levels of the CC and TT genotype of the *FOXO3a* rs4946936 gene variant were −320.80 Kcal/mol and −301.10 Kcal/mol, respectively, indicating that the CC genotype had lower free energy levels. ^21^ Low free energy levels were associated with the mRNA structure and translation, and the changes of mRNA translation at the 5′ and 3′ UTR locations were correlated to affect protein synthesis. ^21^^–^^23^ This explanation might describe the possible underlying mechanism that the CC genotype of the *FOXO3a* rs4946936 gene variant had an association with increased levels of *FOXO3a* in the case of CGL compared to the CT and TT genotype. Moreover, previous study also supported our findings. They found that elevated levels of *FOXO3a* was associated with treatment failure of CGL patient treated with imatinib mesylate, suggesting that the levels of *FOXO3a* might play an important role to contribute the progression of CGL. ^16^ Furthermore, studies also found that elevated levels of *FOXO3a* was associated with adverse prognosis of leukemia. ^24^^,^^25^ However, gene – environment interaction should also be considered to affect the disease progression since it was proven that the FOXO transcription factors had various impact in different population, for example: the role of FOXO transcription factors were found inconclusive to affect the human longevity in the population of Japan, Germany, Italia, and Indonesia. ^26^^,^^27^

To the best of our knowledge, our study is the first to report the association between the levels of *FOXO3a* and the gene polymorphism of *FOXO3a* rs4946936 in the case of CGL patients. Our findings may serve as an initial investigation regarding the role of *FOXO3a* rs4946936 gene polymorphism in affecting the levels of *FOXO3a*, compared to different findings in the case of COPD and vitiligo. In the future, our results may contribute to a better understanding regarding the role of *FOXO3a* rs4946936 gene polymorphism and the levels of *FOXO3a* in the pathogenesis of CGL patients. However, understanding the role of genetics in a disease is problematic and further studies involving gene-gene, gene-environment, and gene-disease interaction are warranted to determine the holistic role of *FOXO3a* gene polymorphism in the pathogenesis of CGL.

Several pertinent limitations to this study should be assessed. First, several factors that might contribute to the severity of CGL in patients including smoking, physical activity, dietary factors, the history of previous medication, and the history of radiation were not analyzed. Second, the small sample size in our study should be interpreted with caution due to the possibility of false positive results. Third, the short time period of the study might not be sufficient to define the real correlation.

## Conclusion

Our study indicates that the CC genotype of the *FOXO3a* rs4946936 gene variant is associated with elevated levels of *FOXO3a.* Our findings may be used as an initial investigation and may provide a better understanding of the role of the *FOXO3a* rs4946936 gene variant in the pathogenesis of CGL.

## Data availability

### Underlying data

Figshare: Underlying data for ‘The association between *FOXO3a* rs4946936 gene polymorphism and the levels of *FOXO3a* among chronic granulocytic leukemia patients treated with imatinib mesylate’, https://doi.org/10.6084/m9.figshare.16529160. ^17^

This project contains the following underlying data:

• Data file.xlsx
• Gel electrophoresis.pdf

## Reporting guidelines

Figshare: STROBE checklist for ‘The association between *FOXO3a* rs4946936 gene polymorphism and the levels of *FOXO3a* among chronic granulocytic leukemia patients treated with imatinib mesylate’, https://doi.org/10.6084/m9.figshare.16529160. ^17^

Data are available under the terms of the Creative Commons Attribution 4.0 International license (CC-BY 4.0)

## Consent

Written informed consent for publication of the patients’ details was obtained from the patients.
