# Supplementary material for: The association between FOXO3a rs4946936 gene polymorphism and the levels of FOXO3a among chronic granulocytic leukemia patients treated with imatinib mesylate
Source: F1000Res. 2022 Apr 11;10:1003. Originally published 2021 Oct 4. [Version 3] doi: 10.12688/f1000research.73054.3 (PMC9005988; doi:10.12688/f1000research.73054.3)
Supplement: Peer review history

# Peer review: Reviewer response for version 3

**DOI:** 10.5256/f1000research.126363.r134582

**Published:** 2022-04-12

**Authors:** Kazuhito Naka

**Affiliations:** 1 Department of Stem Cell Biology, Research Institute for Radiation Biology and Medicine, Hiroshima University, Hiroshima, Japan

Thank you very much for giving me the opportunity to review the revised manuscript to “F1000Research”. I feel that the authors well answered my concern. Thus, I agree to accept this paper to be indexed on “F1000Research”.

Is the work clearly and accurately presented and does it cite the current literature?

Partly

If applicable, is the statistical analysis and its interpretation appropriate?

I cannot comment. A qualified statistician is required.

Are all the source data underlying the results available to ensure full reproducibility?

No source data required

Is the study design appropriate and is the work technically sound?

Yes

Are the conclusions drawn adequately supported by the results?

Yes

Are sufficient details of methods and analysis provided to allow replication by others?

Yes

Reviewer Expertise:

NA

I confirm that I have read this submission and believe that I have an appropriate level of expertise to confirm that it is of an acceptable scientific standard.

# Peer review: Reviewer response for version 2

**DOI:** 10.5256/f1000research.121907.r125948

**Published:** 2022-04-07

**Authors:** Kazuhito Naka

**Affiliations:** 1 Department of Stem Cell Biology, Research Institute for Radiation Biology and Medicine, Hiroshima University, Hiroshima, Japan

While the authors improved the description, they have not corrected the introduction yet. The authors described that “FOXO3a plays a dominant role in carcinogenesis including cell cycle progression, proliferation, the activation of reactive oxygen species, DNA damage, apoptosis, and tumorigenesis." However, it has been known that FOXO3a is implicated in “cell cycle arrest”, “detoxification of reactive oxygen species”, and “DNA damage repair”. Thus, please correct the descriptions.

Is the work clearly and accurately presented and does it cite the current literature?

Partly

If applicable, is the statistical analysis and its interpretation appropriate?

I cannot comment. A qualified statistician is required.

Are all the source data underlying the results available to ensure full reproducibility?

No source data required

Is the study design appropriate and is the work technically sound?

Yes

Are the conclusions drawn adequately supported by the results?

Yes

Are sufficient details of methods and analysis provided to allow replication by others?

Yes

Reviewer Expertise:

Tyrosine kinase inhibitor (TKI) resistant relapse of chronic myelogenous leukaemia (CML) patients.

I confirm that I have read this submission and believe that I have an appropriate level of expertise to confirm that it is of an acceptable scientific standard, however I have significant reservations, as outlined above.

# Response

**Published:** 2022-04-08

**Authors:** Jonny Fajar

**Affiliations:** Brawijaya Internal Medicine Research Center, Department of Internal Medicine, Faculty of Medicine, Universitas Brawijaya, Malang, Indonesia, Indonesia

1. While the authors improved the description, they have not corrected the introduction yet. The authors described that “FOXO3a plays a dominant role in carcinogenesis including cell cycle progression, proliferation, the activation of reactive oxygen species, DNA damage, apoptosis, and tumorigenesis." However, it has been known that FOXO3a is implicated in “cell cycle arrest”, “detoxification of reactive oxygen species”, and “DNA damage repair”. Thus, please correct the descriptions..

Response: We have revised the above description.

# Peer review: Reviewer response for version 2

**DOI:** 10.5256/f1000research.121907.r125947

**Published:** 2022-03-02

**Authors:** Mardiah Hardianti

**Affiliations:** 1 Division of Hematology and Medical Oncology, Department of Internal Medicine, Faculty of Medicine, Public Health and Nursing, Dr. Sardjito Hospital, Universitas Gadjah Mada, Yogyakarta, Indonesia

I found the responses from the authors were sufficient to answer all our questions. I hereby agree for the work to be indexed without any further reservation.

Is the work clearly and accurately presented and does it cite the current literature?

Yes

If applicable, is the statistical analysis and its interpretation appropriate?

Yes

Are all the source data underlying the results available to ensure full reproducibility?

Yes

Is the study design appropriate and is the work technically sound?

Yes

Are the conclusions drawn adequately supported by the results?

Yes

Are sufficient details of methods and analysis provided to allow replication by others?

Yes

Reviewer Expertise:

NA

I confirm that I have read this submission and believe that I have an appropriate level of expertise to confirm that it is of an acceptable scientific standard.

# Peer review: Reviewer response for version 1

**DOI:** 10.5256/f1000research.76675.r120814

**Published:** 2022-02-10

**Authors:** Mardiah Hardianti

**Affiliations:** 1 Division of Hematology and Medical Oncology, Department of Internal Medicine, Faculty of Medicine, Public Health and Nursing, Dr. Sardjito Hospital, Universitas Gadjah Mada, Yogyakarta, Indonesia

1. The idea of this paper is original and highly appreciated. It aimed to elucidate one of the important molecular mechanism of CGL by assessing the target gene polymorphism and its protein FOXO3a. The finding that CC genotype was related to the higher level of FOXO3a which might be related with disease mechanism was explained well by referring to other disease mechanism such as vitiligo and COPD. However, it is still suggested to be explained more in the aspect of molecular mechanism of hematological malignancy. 
2. Other possible explanations for the uninvolved role of CT or TT polymorphisms in CGL disease mechanism should be explained more rather than just comparing their biochemical aspect to the CC phenotype which stated that CC phenotype produced higher level of FOXO3a protein. Any technical or sampling issues could also be other explanations for this finding. 
3. Is there any possible extrapolation for disease mechanism from Indonesian population regarding CC phenotype findings in this study? Please explain either when the answer is yes or no. 

Is the work clearly and accurately presented and does it cite the current literature?

Yes

If applicable, is the statistical analysis and its interpretation appropriate?

Yes

Are all the source data underlying the results available to ensure full reproducibility?

Yes

Is the study design appropriate and is the work technically sound?

Yes

Are the conclusions drawn adequately supported by the results?

Yes

Are sufficient details of methods and analysis provided to allow replication by others?

Yes

Reviewer Expertise:

Molecular mechanism of cancer

I confirm that I have read this submission and believe that I have an appropriate level of expertise to confirm that it is of an acceptable scientific standard, however I have significant reservations, as outlined above.

# Response

**Published:** 2022-02-27

**Authors:** Jonny Fajar

**Affiliations:** Brawijaya Internal Medicine Research Center, Department of Internal Medicine, Faculty of Medicine, Universitas Brawijaya, Malang, Indonesia, Indonesia

**Reviewer2**

 1. The idea of this paper is original and highly appreciated. It aimed to elucidate one of the important molecular mechanism of CGL by assessing the target gene polymorphism and its protein FOXO3a. The finding that CC genotype was related to the higher level of FOXO3a which might be related with disease mechanism was explained well by referring to other disease mechanism such as vitiligo and COPD. However, it is still suggested to be explained more in the aspect of molecular mechanism of hematological malignancy.

 Response: The additional explanation on the possible role of FOXO3a in the context of hematological malignancy has been provided.

 2. Other possible explanations for the uninvolved role of CT or TT polymorphisms in CGL disease mechanism should be explained more rather than just comparing their biochemical aspect to the CC phenotype which stated that CC phenotype produced higher level of FOXO3a protein. Any technical or sampling issues could also be other explanations for this finding.

 Response: The explanation regarding the possible reason why CC genotype may associate with the higher production of FOXO3a levels compared to CT and TT genotypes has been provided in the discussion paragraph 2 (the possible association between the genotype and free energy levels).

 3. Is there any possible extrapolation for disease mechanism from Indonesian population regarding CC phenotype findings in this study? Please explain either when the answer is yes or no.

 Response: The additional explanation on the possible gene – environment interaction including Indonesian population has been provided.

# Peer review: Reviewer response for version 1

**DOI:** 10.5256/f1000research.76675.r118690

**Published:** 2022-01-13

**Authors:** Kazuhito Naka

**Affiliations:** 1 Department of Stem Cell Biology, Research Institute for Radiation Biology and Medicine, Hiroshima University, Hiroshima, Japan

In the valuable manuscript, the authors investigated association between FOXO3a polymorphism and the levels of FOXO3a in serum from chronic granulocytic leukemia (CGL) patients after treatment with imatinib mesylate. They indicated that the CC, CT, and TT genotypes of the FOXO3a rs4946936 gene polymorphism were 35.0%, 48.3%, and 16.7%, respectively. Interestingly, the levels of FOXO3a were elevated in the CGL patients with CC genotype of the FOXO3a rs4946936 gene than those with the CT and TT genotypes. Collectively, the authors concluded that the CC genotype of the FOXO3a rs4946936 gene polymorphism affects the elevated levels the FOXO3a in CGL patients treated with imatinib mesylate.

**Major points**

 In table 2, it is difficult to understand what it means, while it indicates important findings in this study. Probably, first “Mean” reveals the expression levels of FOXO3a. But, I cannot understand the second “Mean”. Please explain each “Mean”. I strongly recommend that the authors reveal the data as “bar graphs” rather than table. It helps the understanding of a wide variety of readers.

 I understood the authors' findings that the CC genotype of the FOXO3a rs4946936 gene polymorphism appears to be correlated with the expression of FOXO3a in the serum of CGL patients treated with imatinib mesylate. However, the authors should indicate clinical significance of the genotype. For example, they should compare the therapeutic effects of imatinib mesylate between the CC genotype and CT and TT genotypes, such as IS of BCR-ABL1 or deep molecular response (DMR) patients. They should also indicate whether or not the levels of FOXO3a in the CC genotypes are elevated post-imatinib administration than pre-imatinib therapy. These data may contribute to predict the therapeutic effect of imatinib mesylate in the patients.

**Minor point**

 In the introduction, the authors described that “FOXO3a plays a dominant role in carcinogenesis including cell cycle progression, proliferation, the activation of reactive oxygen species, DNA damage, apoptosis, and tumorigenesis." However, it has been known that FOXO3a is implicated in “cell cycle arrest”, “detoxification of reactive oxygen species”, and “DNA damage repair”. Thus, please correct the descriptions.

Is the work clearly and accurately presented and does it cite the current literature?

Partly

If applicable, is the statistical analysis and its interpretation appropriate?

I cannot comment. A qualified statistician is required.

Are all the source data underlying the results available to ensure full reproducibility?

No source data required

Is the study design appropriate and is the work technically sound?

Yes

Are the conclusions drawn adequately supported by the results?

Yes

Are sufficient details of methods and analysis provided to allow replication by others?

Yes

Reviewer Expertise:

Tyrosine kinase inhibitor (TKI) resistant relapse of chronic myelogenous leukaemia (CML) patients.

I confirm that I have read this submission and believe that I have an appropriate level of expertise to confirm that it is of an acceptable scientific standard, however I have significant reservations, as outlined above.

# Response

**Published:** 2022-02-27

**Authors:** Jonny Fajar

**Affiliations:** Brawijaya Internal Medicine Research Center, Department of Internal Medicine, Faculty of Medicine, Universitas Brawijaya, Malang, Indonesia, Indonesia

**Reviewer1**

 1. In table 2, it is difficult to understand what it means, while it indicates important findings in this study. Probably, first “Mean” reveals the expression levels of FOXO3a. But, I cannot understand the second “Mean”. Please explain each “Mean”. I strongly recommend that the authors reveal the data as “bar graphs” rather than table. It helps the understanding of a wide variety of readers.

 Response: We have provided Figure 2 instead of Table 2, as suggested by reviewer.

 2. I understood the authors' findings that the CC genotype of the FOXO3a rs4946936 gene polymorphism appears to be correlated with the expression of FOXO3a in the serum of CGL patients treated with imatinib mesylate. However, the authors should indicate clinical significance of the genotype. For example, they should compare the therapeutic effects of imatinib mesylate between the CC genotype and CT and TT genotypes, such as IS of BCR-ABL1 or deep molecular response (DMR) patients. They should also indicate whether or not the levels of FOXO3a in the CC genotypes are elevated post-imatinib administration than pre-imatinib therapy. These data may contribute to predict the therapeutic effect of imatinib mesylate in the patients.

 Response: In our present study, we did not compare the levels of FOXO3 between pre and post imatinib treatment. Therefore, we could not provide the holistic information regarding those comparison. However, we have added the additional discussion about the proposed effect of FOXO3a levels and the treatment of imatinib.

 3. In the introduction, the authors described that “FOXO3a plays a dominant role in carcinogenesis including cell cycle progression, proliferation, the activation of reactive oxygen species, DNA damage, apoptosis, and tumorigenesis." However, it has been known that FOXO3a is implicated in “cell cycle arrest”, “detoxification of reactive oxygen species”, and “DNA damage repair”. Thus, please correct the descriptions.

 Response: The above description has been revised.
